# The efficacy of the *Kampo* medicine *rikkunshito* for chemotherapy-induced anorexia (RICH trial): study protocol for a randomized controlled trial

**DOI:** 10.1186/s13063-017-2227-6

**Published:** 2017-10-18

**Authors:** Takuya Inoue, Hironori Takagi, Yuki Owada, Yuzuru Watanabe, Takumi Yamaura, Mitsuro Fukuhara, Satoshi Muto, Naoyuki Okabe, Yuki Matsumura, Takeo Hasegawa, Jun Osugi, Mika Hoshino, Mitsunori Higuchi, Yutaka Shio, Hiroshi Yokouchi, Kenya Kanazawa, Katsuya Ohbuchi, Takahisa Fukushima, Mitsuru Munakata, Hiroyuki Suzuki

**Affiliations:** 1 Department of Chest Surgery, Fukushima Medical University School of Medicine, 1 Hikarigaoka, Fukushima, 960-1295 Japan; 2 Department of Chest Surgery, Takeda General Hospital, Fukushima, Japan; 3 Department of Respiratory Medicine, Fukushima Medical University School of Medicine, Fukushima, Japan; 4 Kampo Research & Development Division, Tsumura & Co., Tokyo, Japan

**Keywords:** Complementary medicine, Lung cancer, Oncology, Medical management, *Kampo* medicine, Rikkunshito, Cisplatin

## Abstract

**Background:**

Cisplatin is a key drug in lung cancer therapy. However, cisplatin is also well known to induce gastrointestinal disorders, such as chemotherapy-induced nausea and vomiting, anorexia, and weight loss. These symptoms sometimes affect patients’ quality of life and make continuation of chemotherapy difficult. Anorexia is a cause of concern for patients with cancer because a persistent loss of appetite progresses to cancer cachexia. Although evidence-based management for chemotherapy has recently been established, there is room for improvement.

**Methods/design:**

This placebo-controlled, double-blind, randomized trial will aim to determine the efficacy of the traditional Japanese *Kampo* medicine *rikkunshito* (TJ-43) for preventing anorexia caused by cisplatin-including chemotherapy in patients with lung cancer. Patients with lung cancer who plan to receive cisplatin-including chemotherapy will be recruited. Patients who provide written consent will be randomly allocated to receive either TJ-43 (arm A) or placebo (arm B) for one course of chemotherapy (21 or 28 consecutive days). Investigators and patients will be masked to the treatment assignment throughout the trial. The primary endpoint will be evaluated as the change in dietary intake from day 0 (the day before the start of chemotherapy) to day 7 of cisplatin-including chemotherapy. The two arms of the trial will comprise 30 patients each. From November 2014, a total of 60 patients will be recruited, and recruitment for the study is planned to be complete by October 2017.

**Discussion:**

This trial is designed to examine the efficacy of *rikkunshito* (TJ-43) for reducing anorexia and maintaining food intake caused by cisplatin-including chemotherapy in patients with lung cancer.

**Trial registration:**

Japan Pharmaceutical Information Center Clinical Trials Information (JAPIC CTI), trial registration: JAPIC CTI-142747. Registered on 15 December 2014; the RICH trial.

**Electronic supplementary material:**

The online version of this article (doi:10.1186/s13063-017-2227-6) contains supplementary material, which is available to authorized users.

## Background

Cisplatin is a key drug in lung cancer chemotherapy. However, cisplatin is also well known to induce gastrointestinal disorders, such as chemotherapy-induced nausea and vomiting (CINV), anorexia, and weight loss. These symptoms sometimes affect patients’ quality of life (QOL) and make continuation of chemotherapy difficult. Although the mechanism of chemotherapy-induced appetite loss is not thoroughly understood, cisplatin-related acute gastrointestinal disorders reportedly involve secretion of serotonin (5-hydroxytryptamine, 5-HT) from enterochromaffin cells. Therefore, 5-HT3 receptor antagonists are widely used to prevent cisplatin-induced nausea and vomiting [1]. Evidence-based CINV management has recently been established by several societies, including the National Comprehensive Cancer Network [2], American Society of Clinical Oncology [3], and Multinational Association of Supportive Care in Cancer/European Society for Medical Oncology [4]. Furthermore, since guidelines for the proper use of antiemetics were announced by the Japan Society of Clinical Oncology in May 2010 [5], the management of CINV in Japan has steadily improved [6]. According to the above guidelines, cisplatin is classified as highly emetogenic chemotherapy (HEC), and the standard prophylaxis for CINV is a three-drug combination of a 5-HT3 receptor antagonist, neurokinin-1 receptor antagonist, and dexamethasone. In addition, a phase III randomized controlled trial [7], which formed the basis of the current standard supportive care for HEC, reported an approximately 80% complete remission rate of acute-onset CINV (≤24 h after anti-cancer agent administration) and a 60 to 70% complete remission rate of late-onset CINV (24–120 h after anti-cancer agent administration).

The above-mentioned antiemetic agents that have been introduced as treatment for CINV have led to improvements in CINV management. However, there is room for further improvement in the management of chemotherapy, especially HEC. Anorexia is a cause of concern for patients with cancer because a persistent loss of appetite progresses to cancer cachexia. In patients treated with cisplatin, anorexia is thought to be caused not only by CINV but also by changes in ghrelin dynamics. Ghrelin is an intense appetite-enhancing hormone comprising 28 amino acids and is secreted mainly from the stomach. The plasma ghrelin concentration is thought to be related to gastrointestinal disorders [8].

*Kampo* medicines are produced uniquely in Japan and have been approved by the Ministry of Health, Labor and Welfare of Japan for treatment of numerous diseases. *Rikkunshito* (TJ-43) is a traditional Japanese *Kampo* medicine that has been prescribed to treat upper gastrointestinal symptoms such as appetite loss and nausea. It comprises eight herbal components, containing a dried extract of the following: 19% *Atractylodes lancea* rhizome, 19% ginseng (*Ginseng radix*), 19% *Pinellia* tuber (*Pinellia ternata*), 19% *Poria sclerotium* (hoelen), 9% jujube (*Ziziphus fructus*), 9% *Citrus unshiu* peel (*Aurantii nobilis pericarpium*), 5% glycyrrhiza (*Glycyrrhizae radix*), and 2% ginger (*Zingiber* rhizome).

Previous studies have reported that TJ-43 enhances digestive tract motility [9], improves the gastric accommodation reflex [10], protects against gastric mucosal injury [11], and enhances appetite [12]. Based on these mechanisms, TJ-43 has been used to treat various gastrointestinal tract diseases, such as functional dyspepsia [13], gastroesophageal reflux disease [14], and chemotherapy-induced nausea [15].

We previously conducted a retrospective analysis of the efficacy of TJ-43 for preventing CINV and maintaining food intake in patients with lung cancer treated with cisplatin-based chemotherapy (presented at the 113rd Annual Meeting of the Japan Surgical Society, not published). Patients receiving chemotherapy for treatment of lung cancer were divided into those using only conventional antiemetic agents and those receiving chemotherapy to which TJ-43 was added. We showed that the rate of late-onset CINV as assessed by the Multinational Association of Supportive Care in Cancer questionnaire [16] was 76% in patients receiving TJ-43 and 40% in patients not receiving TJ-43. In addition, the average dietary intake rate from day 1 (the start date of chemotherapy) to day 5 was 72.2 ± 18.1% (mean ± standard deviation) in patients receiving TJ-43 and 58.4 ± 18.2% in patients not receiving TJ-43.

We are, therefore, conducting a placebo-controlled, double-blind, randomized trial to evaluate the efficacy of TJ-43 for chemotherapy-induced anorexia (RICH trial). We herein report the details of the trial design. This trial will be carried out in compliance with the ordinance of the Ministry of Health, Labor and Welfare in Japan, Good Post-Marketing Study Practice, Good Clinical Practice, and the guideline for clinical trials “Statistical Principles for Clinical Trials” [17]. This trial was also planned with reference to “Structure and Content of Clinical Study Reports” [18].

## Methods/design

### Objectives

**Fig. 1:**
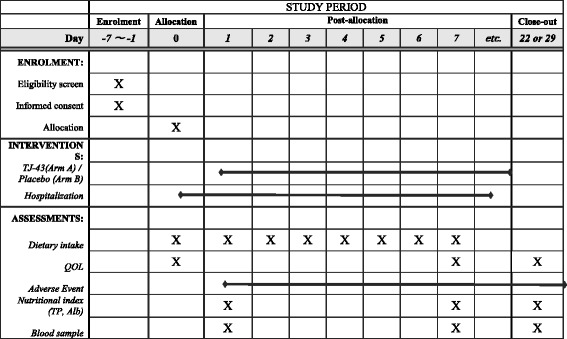
Summary of enrollment, interventions, assessments, and timing for measurements (Standard Protocol Items: Recommendations for Interventional Trials (SPIRIT) Figure)

The RICH trial is a placebo-controlled, double-blind, randomized trial that aims to examine the efficacy of TJ-43 for anorexia and maintaining food intake in patients with lung cancer who plan to receive cisplatin-including chemotherapy. The Standard Protocol Items: Recommendations for Interventional Trials (SPIRIT) Figure is shown in Fig. 1.

### Endpoints

#### Primary endpoint

The primary endpoint is:

- Change in dietary intake from day 0 (the day before the start of chemotherapy) to day 7 of chemotherapy

#### Secondary endpoints

The secondary endpoints are:

- Changes in QOL assessment (QLQ-C30 [19] + QLQ-LC13 [20]) at days 0, 7, and 22 or 29 (the start date of the next chemotherapy course)
- The incidence of any adverse events, except anorexia
- The proportion of patients who receive dose-reduced cisplatin-including chemotherapy in the next course
- The proportion of patients who delay the start of cisplatin-including chemotherapy in the next course
- The proportion of patients requiring extended hospitalization
- Changes in nutritional indices (total protein and albumin concentrations) at days 1, 7, and 22 or 29 (the start date of the next chemotherapy course)

#### Other outcome measures

Other outcome measures are:

- Changes in plasma metabolite concentrations at days 1, 7, and 22 or 29 (the start date of the next chemotherapy course)

### Eligibility criteria

#### Inclusion criteria

The inclusion criteria are:

- Patients with a histologically or cytologically confirmed diagnosis of lung cancer
- Patients planning to receive cisplatin-including chemotherapy
- Patients capable of oral ingestion of medicine
- Adequate function of vital organs and bone marrow
- Age of 20 years or older at the time of registration
- Eastern Cooperative Oncology Group performance status of 0 or 1
- Written informed consent provided to participate in the study

#### Exclusion criteria

The exclusion criteria are:

- The use of any *Kampo* medicines within 2 weeks before registration
- Hemoptysis (> 2.5 mL/day) within 2 weeks before registration
- Peripheral neuropathy of grade > 1 (National Cancer Institute Common Terminology Criteria for Adverse Events version 4.0)
- Brain metastasis
- Pregnant or lactating women
- Current treatment with cisplatin-including chemotherapy
- Patients scheduled to receive cisplatin (≤ 20 mg/m^2^)
- Serious infection
- Severe complications (e.g., cardiac disease, interstitial pneumonia, uncontrolled diabetes mellitus)
- Uncontrolled pleural effusion, ascites or pericardial effusion
- Other active malignancies
- History of severe drug allergy
- Any other medical condition that makes the patient unsuitable for inclusion in the study, according to the opinion of the investigator

### Registration

An eligibility report form will be sent to the Registration Center at Tsumura & Co. (Tokyo, Japan). Patients who meet all eligibility criteria will be randomly allocated to receive either TJ-43 (arm A) or placebo (arm B) at the individual institutions. Each participant must be hospitalized to receive chemotherapy. The placebo is indistinguishable from the TJ-43 in appearance. Investigators prescribing either TJ-43 or placebo to each patient will not know which patient is allocated to which arm.

Patients and investigators (including the investigators assessing blood tests) will be masked to the treatment assignment throughout the study. Each arm will comprise 30 patients. Recruitment began in November 2014 and will end in October 2017 or until 60 participants have been recruited.

This trial is being conducted at two institutions in Fukushima prefecture of Japan.

### Participating institutions

This trial is being conducted at Fukushima Medical University and Takeda General Hospital, Fukushima, Japan

### Treatment methods

#### Study drug

The study drug (TSUMURA *Rikkunshito* Extract Granules for Ethical Use or placebo) will be administered at a dose of 2.5 g three times per day (total daily dose of 7.5 g). The placebo formulation matches that of TJ-43 in terms of appearance and texture. TJ-43 and the matching placebo were manufactured by Tsumura & Co. (Tokyo, Japan).

#### Arm A (TJ-43 group) and arm B (placebo group)

**Fig. 2:**
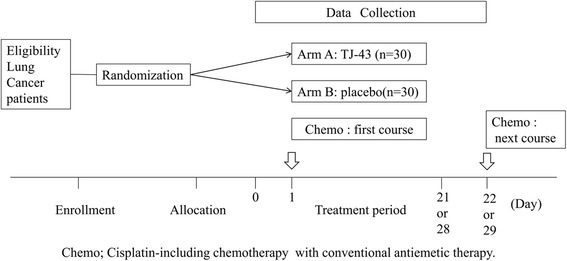
Overview of the RICH trial procedure

TJ-43 (arm A) or placebo (arm B) will be administered per os at a dose of 2.5 g three times per day (total daily dose of 7.5 g), either before meals or between meals, for one course of chemotherapy (21 or 28 consecutive days). The study drug will be initiated on the first day of chemotherapy (day 1) and continued until the day before the start of the next course of chemotherapy (day 22 or 29; allowable range of + 7 days) (Fig. 2). If the timing of the next chemotherapy administration is postponed, the investigators will continue to administer the study drug for the extended period.

The investigators will prescribe the study drug. Both the participants and investigators will be blinded to the identity of the drug (TJ-43 or placebo).

Patients in both arms will also receive conventional antiemetic therapy, i.e., palonosetron (0.75 mg on day 1 administered intravenously), dexamethasone (9.9 mg on day 1 administered intravenously, 8 mg on days 2–4 administered per os), and aprepitant (125 mg on day 1 and 80 mg on days 2–3 administered per os), in accordance with established guidelines [5].

### Randomization and blinding

The participants will be assigned to either the TJ-43 group or the placebo group in a 1:1 ratio using a block randomization method. A randomization list has been pregenerated, and this assignment list will be kept in a safe until unblinding. The randomization list will not be disclosed to the investigators. The placebo drug is indistinguishable from TJ-43 in appearance. Therefore, both the investigators and participants will be blinded to the treatment group throughout the study. Unblinding will take place only after the trial is finished and data are fixed, except in the case of emergencies.

### Prohibited drugs

The concomitant intake of other *Kampo* medicines or other medicaments comprising a component of TJ-43 is prohibited during the administration of TJ-43 or placebo to avoid interactions or effects on the evaluation of drug efficacy.

### Criteria for discontinuing the protocol treatment

The following will be considered criteria for discontinuing the protocol treatment:

- Consent is withdrawn
- Investigators judge that it is difficult to continue the test because of deterioration of the patient’s primary disease
- Investigators judge that it is difficult to continue the test because of adverse events
- Investigators judge that it is difficult to continue the test because of the development of severe diseases other than lung cancer
- The patient is deemed ineligible for the trial after registration
- The patient’s medication compliance is inadequate and does not improve despite medication instruction after registration
- The patient discontinues the medication at their own discretion
- The patient fails to visit the hospital after the trial has begun
- Investigators judge that it is suitable to discontinue the test

### Data collection

After registration and assignment to treatments, prospective data will be collected for all patients, including sex, date of birth, physical findings, and clinical information (such as medical history, complications, and regimen of lung cancer chemotherapy). The investigators will measure the patients’ dietary intake during each meal from day 0 (the day before the start of chemotherapy) to day 7. For each meal, the weight of food (including tableware) before and after the meal will be measured. Dietary intake will be calculated by subtracting the weight of food consumed (including tableware) after the meal from that of food (including tableware) before the meal. The investigators will also check meals other than hospital diets.

In addition, the investigators will check changes in the patients’ QOL assessment at days 0, 7, and 22 or 29 (the start date of the next chemotherapy course), the incidence of any adverse events except anorexia, the proportion of patients who received dose-reduced cisplatin-including chemotherapy in the next course, the proportion of patients who delayed the start of cisplatin-including chemotherapy in the next course, and the proportion of patients requiring extended hospitalization.

Blood samples will be routinely collected at days 1, 7, and 22 or 29. Two sets of specimens will be drawn: routine monitoring samples and trial samples. Possible side effects, such as neutropenia and renal dysfunction, will be checked in both groups. The routine blood samples will undergo measurements of the hemoglobin concentration; leukocyte, neutrophil, and platelet counts; and urea nitrogen and creatinine concentrations. These parameters will be used to manage the side effects of lung cancer treatment in daily clinical practice.

Possible side effects in the TJ-43 group also include liver dysfunction, myopathy, hypersensitivity, and electrolyte abnormalities. The trial blood samples will be collected not only to examine safety (liver enzyme, creatinine phosphokinase, and electrolyte concentrations) but also to examine nutritional indices (total protein and albumin concentrations).

Japanese *Kampo* medicine is often used to manage the side effects of lung cancer treatment, although it is not necessarily used every time. Japanese *Kampo* medicine is prescribed on the unique basis of *Kampo* diagnosis, the concept of which differs from that of Western medicine. Because the concept of *Kampo* diagnosis has not yet been fully elucidated, it is necessary to explore the biomarkers reflecting the *Kampo* diagnosis and predicting the effect. Metabolomic analysis was recently utilized in clinical studies to explore biomarkers. Because metabolites are located downstream of deoxyribonucleic acid (DNA), ribonucleic acid (RNA), and proteins, metabolomics is closer to the phenotype than are other “omics.” Therefore, we will plan to explore various biomarkers to predict the effect and side effect of TJ-43 using plasma metabolomic analysis.

We will measure QOL using two self-administered QOL questionnaires (the QLQ-C30 and QLQ-LC13) at days 0, 7, and 22 or 29 (the start date of the next chemotherapy course).

### Data management

The investigators and clinical research coordinator will maintain individual records for each patient, such as the informed consent document, medical record, laboratory data, and Case Report Form, according to Good Clinical Practice. The investigators will record adverse effects in the patients’ Case Report Forms. Adverse events will be followed up continually until resolution. The investigators will be responsible for oversight of the data management of the trial. An independent Data Monitoring Committee will not be established. Instead, an independent medical advisor has been set up and will regularly assess safety, feasibility or any other problems. Data and all appropriate documentation will be stored for a minimum of 5 years after completion of the trial.

### Sample size

The sample size was calculated based on our retrospective data showing that the average dietary intake rate from day 1 to 5 was 72.2% ± 18.1% (mean ± standard deviation) in patients administered TJ-43 and 58.4% ± 18.2% in patients who were not administered TJ-43 (unpublished data). Based on this preliminary study, we calculated that 28 patients per group would be required to achieve a power of 80% with a two-sided significance level of *p* < 0.05 for detecting the superiority of concomitant treatment with TJ-43 by a *t* test. Here, the assumed effect size (*d*) was 0.76. To account for possible dropouts (10%), the target number of patients was, therefore, set at 30 per group (60 in total) (see Additional file 1).

### Study design and statistical analysis

The effects of TJ-43 against chemotherapy-induced anorexia have yet to be determined. The RICH trial is designed to evaluate the efficacy of TJ-43 for anorexia in patients with lung cancer undergoing treatment with cisplatin-including chemotherapy. It is a placebo-controlled, double-blind, randomized trial that will be conducted at two institutions.

The primary endpoint will be the change in dietary intake from day 0 (the day before the start of chemotherapy) to day 7. The mean dietary intake in hospital (MDIH) is defined as the total hospital diet intake divided by the number of records. The investigators will calculate the MDIH each day from day 0 through day 7 for each patient, and the value at day 0 will be used as the baseline. The primary endpoint variable of this trial is the mean change in the MDIH from the baseline, averaged from day 1 through day 7. This will be evaluated by analysis of covariance (ANCOVA), which employs the baseline value as the covariate, to estimate the difference between the two treatment groups (*n* = 30 per group). Additionally, for ease of interpretation, the time profile of MDIH from day 1 through day 7 will be analyzed by a linear mixed-effects model using the baseline value as the covariate.

The secondary endpoints, namely the changes in nutritional indices (total protein and albumin concentrations), will also be analyzed by ANCOVA, which employs the baseline value (day 1) as the covariate. The changes in QOL assessment (QLQ-C30, QLQ-LC13) will be analyzed by ANCOVA for each item, which employs the baseline value (day 0) as the covariate. Items in the QOL assessment include the evaluation of nausea, vomiting, and anorexia. In addition, the proportion of patients who receive dose-reduced or delayed chemotherapy and the proportion of patients requiring extended hospitalization will be compared between the two treatment groups using the chi-square test or Fisher’s exact test. The incidence of adverse event will be compared by means of Fisher’s exact test.

The analyses of efficacy outcomes will be performed in the full analysis set. An interim analysis will not be performed.

This trial will be conducted in accordance with the World Medical Association Declaration of Helsinki. The protocol has been approved by the Institutional Review Board of each participating institute. Written informed consent will be obtained from all patients before enrollment and randomization by investigators. The SPIRIT Checklist is provided as an Additional file 2.

## Discussion

The aim of this trial is to examine the efficacy of TJ-43 for anorexia and food intake in patients with lung cancer who receive cisplatin-including chemotherapy.

*Kampo* medicines, such as TJ-43, are widely used for numerous diseases in Japan. The drugs are relatively inexpensive and usually used alone or in combination with Western medicine. However, *Kampo* medicines are not necessarily used in every case because the concept of *Kampo* diagnosis is unique and the precise mechanism has not been fully elucidated. TJ-43 has been adapted for anorexia and vomiting within the insurance coverage in Japan. TJ-43 reportedly improves anorexia through ghrelin release [12]. Additionally, in our previous study, TJ-43 was effective for both CINV and maintaining dietary intake in patients with lung cancer who received chemotherapy. This result suggests that TJ-43 might improve anorexia by suppressing CINV, resulting in increased dietary intake.

However, weight loss has also been identified as an independent predictor of short survival for patients with lung cancer receiving chemotherapy [21]. Furthermore, not only ghrelin levels and anorexia but also dietary intake reportedly decreases in patients who receive cisplatin-including chemotherapy [22].

For this reason, we focused on dietary intake and used meal intake as the primary endpoint. In this trial, we are testing only patients with lung cancer who will receive cisplatin; based on the results; however, TJ-43 may also be effective for chemotherapy regimens other than HEC.

The exclusion criteria are limitations to the external validity of the results. Whether the results of this study will apply to other ethnicities is unclear. It seems necessary to perform validation in these groups. We also plan to explore objective biomarkers to predict the effect of TJ-43 using plasma metabolomic analysis.

### Trial status

The RICH study (protocol version 2.0) was registered in the Japan Pharmaceutical Information Center Clinical Trials Information (JAPIC CTI), Japan (registration number: JAPIC CTI-142747) on 15 December 2014. Recruitment started in November 2014 and will end in October 2017 or until 60 participants have been recruited. The first patient was recruited on 20 January 2015. This study is ongoing according to the amended protocol version 2.5 and is currently in the recruitment phase.

## Additional files

Additional file 1: Sample size calculation. (DOCX 23 kb)

Additional file 2: SPIRIT 2013 Checklist: recommended items to address in a clinical trial protocol and related documents*. (DOC 121 kb)

## Abbreviations

5-HT: 5-hydroxytryptamine
ANCOVA: Analysis of covariance
CINV: Chemotherapy-induced nausea and vomiting
HEC: Highly emetogenic chemotherapy
MDIH: Mean dietary intake in hospital
QOL: Quality of life
