# Supplementary material for: The efficacy of the Kampo medicine rikkunshito for chemotherapy-induced anorexia (RICH trial): study protocol for a randomized controlled trial
Source: Trials. 2017 Oct 18;18:485. doi: 10.1186/s13063-017-2227-6 (PMC5648481; doi:10.1186/s13063-017-2227-6)
Supplement: Supplementary file 1 — Sample size calculation. (DOCX 23 kb) [file 13063_2017_2227_MOESM1_ESM.docx]

**Sample size**

The sample size was calculated based on our retrospective data showing that the average dietary intake rate from day 1 to 5 was 72.2% ± 18.1% (mean ± standard deviation) in patients administered TJ-43 and 58.4% ± 18.2% in patients who were not administered TJ-43 (unpublished data). Based on this preliminary study, we calculated that about 28 patients per group would be required to achieve a power of 80% with a two-sided significance level of p < 0.05 for detecting the superiority of concomitant treatment with TJ-43 by a t-test, as the minimum positive integer *n* which satisfies the following formula:

$$\Pr\left(T>q_{1-\alpha/2,2\cdot\left(n-1\right)}\left|\lambda=\frac{\delta}{\sigma}\cdot\sqrt{\frac{n}{2}}\right.\right)+\Pr\left(T<q_{\alpha/2,2\cdot\left(n-1\right)}\left|\lambda=\frac{\delta}{\sigma}\cdot\sqrt{\frac{n}{2}}\right.\right)>1-\beta$$

Here the test statistics $T$is a non-central t-variate with $2\cdot\left(n-1\right)$ degree of freedom and non-centrality parameter$\lambda=\frac{\delta}{\sigma}\cdot\sqrt{\frac{n}{2}}$, and $q_{x,\nu}$ is the $100\cdot x$ percentile of central t distribution with $\nu$ degrees of freedom.

We assumed the effect size ($\delta/\sigma$) 0.76. To account for possible dropouts (10%), the target number of patients was therefore set at 30 per group (60 in total).
